# Transcriptional Regulation of Multi-Drug Tolerance and Antibiotic-Induced Responses by the Histone-Like Protein Lsr2 in M. tuberculosis

**DOI:** 10.1371/journal.ppat.0030087

**Published:** 2007-06-22

**Authors:** Roberto Colangeli, Danica Helb, Catherine Vilchèze, Manzour Hernando Hazbón, Chee-Gun Lee, Hassan Safi, Brendan Sayers, Irene Sardone, Marcus B Jones, Robert D Fleischmann, Scott N Peterson, William R Jacobs, David Alland

**Affiliations:** 1 Division of Infectious Disease and the Center for Emerging Pathogens, Department of Medicine, New Jersey Medical School, University of Medicine and Dentistry of New Jersey, Newark, New Jersey, United States of America; 2 Howard Hughes Medical Institute, Department of Microbiology and Immunology, Albert Einstein College of Medicine, Bronx, New York, United States of America; 3 Department of Biochemistry and Molecular Biology, New Jersey Medical School, University of Medicine and Dentistry of New Jersey, Newark, New Jersey, United States of America; 4 Pathogen Functional Genomics Resource Center, The Institute for Genomic Research, J. Craig Venter Institute, Rockville, Maryland, United States of America; University of Washington, United States of America

## Abstract

Multi-drug tolerance is a key phenotypic property that complicates the sterilization of mammals infected with *Mycobacterium tuberculosis.* Previous studies have established that *iniBAC,* an operon that confers multi-drug tolerance to M. bovis BCG through an associated pump-like activity, is induced by the antibiotics isoniazid (INH) and ethambutol (EMB). An improved understanding of the functional role of antibiotic-induced genes and the regulation of drug tolerance may be gained by studying the factors that regulate antibiotic-mediated gene expression. An M. smegmatis strain containing a *lacZ* gene fused to the promoter of *M. tuberculosis iniBAC* (P*_iniBAC_*) was subjected to transposon mutagenesis. Mutants with constitutive expression and increased EMB-mediated induction of P*_iniBAC_*::*lacZ* mapped to the *lsr2* gene (MSMEG6065), a small basic protein of unknown function that is highly conserved among mycobacteria. These mutants had a marked change in colony morphology and generated a new polar lipid. Complementation with multi-copy *M. tuberculosis lsr2* (Rv3597c) returned P*_iniBAC_* expression to baseline, reversed the observed morphological and lipid changes, and repressed P*_iniBAC_* induction by EMB to below that of the control M. smegmatis strain. Microarray analysis of an *lsr2* knockout confirmed upregulation of *M. smegmatis iniA* and demonstrated upregulation of genes involved in cell wall and metabolic functions. Fully 121 of 584 genes induced by EMB treatment in wild-type M. smegmatis were upregulated (“hyperinduced”) to even higher levels by EMB in the *M. smegmatis lsr2* knockout. The most highly upregulated genes and gene clusters had adenine-thymine (AT)–rich 5-prime untranslated regions. In *M. tuberculosis,* overexpression of *lsr2* repressed INH-mediated induction of all three *iniBAC* genes, as well as another annotated pump, *efpA*. The low molecular weight and basic properties of Lsr2 (pI 10.69) suggested that it was a histone-like protein, although it did not exhibit sequence homology with other proteins in this class. Consistent with other histone-like proteins, Lsr2 bound DNA with a preference for circular DNA, forming large oligomers, inhibited DNase I activity, and introduced a modest degree of supercoiling into relaxed plasmids. Lsr2 also inhibited in vitro transcription and topoisomerase I activity. Lsr2 represents a novel class of histone-like proteins that inhibit a wide variety of DNA-interacting enzymes. Lsr2 appears to regulate several important pathways in mycobacteria by preferentially binding to AT-rich sequences, including genes induced by antibiotics and those associated with inducible multi-drug tolerance. An improved understanding of the role of *lsr2* may provide important insights into the mechanisms of action of antibiotics and the way that mycobacteria adapt to stresses such as antibiotic treatment.

## Author Summary

Understanding the cellular processes stimulated when Mycobacterium tuberculosis is treated with antibiotics may provide clues as to why months of therapy and use of several drugs simultaneously are required to prevent antibiotic resistance. Antibiotic treatment “turns on” or induces certain M. tuberculosis genes. These genes are of special interest because they appear to help M. tuberculosis survive the stress of antibiotic treatment. Our study of the regulation of antibiotic-induced genes, including *iniBAC,* in two mycobacterial species revealed that a small protein called Lsr2 controls *iniBAC* and other antibiotic-induced genes, especially ones related to the cell wall. Lsr2 binds to DNA in a relatively non-specific manner and appears to inhibit certain enzymes that must interact with DNA as part of their function. These properties differentiate Lsr2 from classical regulators of gene expression that bind to specific DNA sequences, and suggest that Lsr2 is a novel histone-like protein. These proteins regulate genes by changing the way DNA is shaped, and, indeed, we found that Lsr2 can change the shape of DNA by introducing a small number of coils into its structure. Our results suggest that Lsr2 is a major regulator of antibiotic-induced responses in mycobacteria.

## Introduction

Mycobacterium tuberculosis appears to generate specific and coordinated transcriptional responses to antibiotic treatment [1,2]. Several broad categories of genes are induced by antibiotics, including a number involved in stress responses and others linked to specific metabolic pathways that are inhibited by antibiotics [1]. The functional roles of antibiotic-induced transcriptional changes are poorly understood. Some changes are likely to be adaptive in that they induce antimicrobial tolerance or are important for intrinsic drug resistance [3–5]. Other changes are likely to be detrimental to the cell and may be ultimately linked to cell death. An improved understanding of the functional role of antibiotic-induced genes may be gained by studying the factors that regulate their expression. Histone-like proteins are reasonable candidates for regulators of antibiotic responses in bacteria because they assist in the control of stationary and exponential phase cell growth and regulate genes that respond to environmental changes [6,7]. Whether histone-like proteins influence the transcriptional response to antibiotics is not known.

The *M. tuberculosis iniBAC* operon (*iniB* or Rv0341, *iniA* or Rv0342, and *iniC* or Rv0343) encodes an important example of antibiotic-regulated genes. This operon is specifically induced by antibiotics such as isoniazid (INH) and ethambutol (EMB) that inhibit cell wall biosynthesis [2], but it is not induced by other stresses, such as hydrogen peroxide and heat shock, or by factors such as lysozyme that digest the cell wall [8]. It has recently been demonstrated that M. bovis BCG strains overexpressing *M. tuberculosis iniA* grow and survive longer than control strains upon exposure to inhibitory concentrations of either INH or EMB, a condition analogous to classical antibiotic tolerance [3].

The goal of the current study was to identify genes required for transcriptional repression of the *iniBAC* promoter (P*_iniBAC_*) and to determine whether this regulatory pathway is part of a broader regulatory network in mycobacteria. Here, we demonstrate that the *lsr2* gene product downregulates transcription of the *M. tuberculosis iniBAC* genes as well as another INH-induced pump, *efpA*. We found that Lsr2 exhibits properties similar to other bacterial histone-like proteins, suggesting that it regulates gene expression by controlling chromosomal topology. This study represents the first report to our knowledge of a gene that regulates antibiotic-induced transcription in M. tuberculosis and suggests that Lsr2 has important regulatory functions in mycobacteria.

## Results

### The *lsr2* Gene Is Required to Repress P*_iniBAC_* Activity

**Figure 1:**
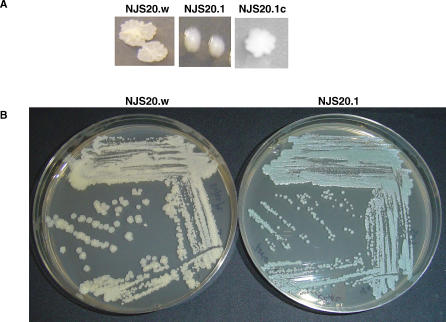
Morphology and β-Lactamase Activity of the *lsr2::Tn5370* Mutant in M. smegmatis (A) Magnification of individual colonies. Left, wild-type NJS20.w (control); center, NJS20.1 (*lsr2* transposon mutant); right, NJS20.1c (complemented *lsr2* transposon mutant). (B) Overall morphology and color of the two strains. Left, NJS20.w; right, NJS20.1

Previous studies have found an association between antibiotic-mediated induction of the *iniBAC* genes and multi-drug tolerance in BCG [3]. We performed transposon mutagenesis studies to identify repressors of *iniBAC* transcription and gain a better understanding of the regulation of drug tolerance. Mutagenesis was performed in NJS20, an M. smegmatis Mc^2^155 strain that contained a single copy of the M. tuberculosis P*_iniBAC_* fused to a *lacZ* reporter inserted into *attP* [8]. NJS20 normally exhibits a subtle light tan-blue color when cultured on media containing X-gal (Figure 1A). We found five strongly blue transposon mutants that had an unusual round and shiny colony morphology (Figure 1A and 1B). This morphology had been previously reported for *lsr2* transposon mutants [9]. Three of the blue colonies were selected and found to contain transposon insertions into two separate sites of the *M. smegmatis lsr2* (MSMEG6056) gene (http://cmr.tigr.org/tigr-scripts/CMR/GenomePage.cgi?org_search =&org=gms).

**Figure 2:**
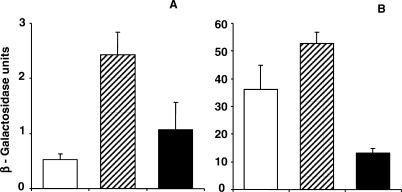
Effect of *lsr2* Gene Inactivation and Overexpression on Activity of the *M. tuberculosis iniBAC* Promoter The activity of the P*_iniBAC_* in the presence or absence of 5 ug/ml EMB is indicated by β-galactosidase units. (A) “Uninduced” cultures without EMB treatment. (B) “Induced” cultures treated with EMB. Open columns, β-galactosidase activity of an NJS20.w control containing a random transposon insertion; dashed columns, the *lsr2* transposon mutant strain NJS20.1; closed columns, the complemented mutant NJS20.1c. The mean and standard deviations of at least triplicate experiments of each strain are shown. Note that different scales were used for β-lactamase units in (A) and (B).

We investigated the role of *lsr2* in repressing basal and antibiotic-induced P*_iniBAC_* activity. An NJS20 *(lsr2::Tn5370) lsr2* transposon mutant (NJS20.1) was cultured to mid log phase, EMB or control media was added to each culture for 24 h, and *lacZ* expression was measured. A second NJS20 strain with an intact *lsr2* gene containing a random transposon insertion (NJS20.w) was selected from the same transposon library to serve as a control. The results of these experiments showed that levels of β-galactosidase activity were approximately five times higher in the NJS20.1 *lsr2* transposon mutant strain than in the control strain in the absence of antibiotic treatment (Figure 2A). P*_iniBAC_* was also induced to higher levels in NJS20.1 than in NJS20.w after treatment with EMB (Figure 2B). The NJS20.1 *lsr2* transposon mutant was then complemented by overexpressing the *M. tuberculosis lsr2* gene (Rv3597c) in pMP167, creating strain NJS20.1c. In the absence of antibiotic treatment, complementation restored the wild-type phenotype and lowered basal *lacZ* expression to levels not significantly different than those of the wild-type NJS20.w control (Figure 2A). The colony morphology also reverted to normal in the complemented strain (Figure 1A). The complemented strain showed significantly less P*_iniBAC_* induction than the NJS20.w transposon mutant control in the presence of EMB treatment. In other words, the presence of *lsr2* on a multi-copy plasmid actually repressed EMB-mediated P*_iniBAC_* induction below that of the wild-type strain (Figure 2B). These results indicate that the *lsr2* gene controls both basal and antibiotic-induced levels of *iniBAC* expression.

### Effect of *lsr2* Deletion and Overexpression on Antibiotic Susceptibility

**Figure 3:**
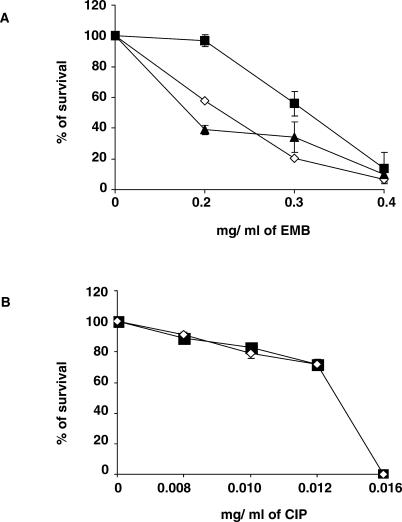
Effect of *lsr2* Inactivation and Overexpression on Antibiotic Susceptibility in M. smegmatis (A and B) Antibiotic susceptibility is calculated by examining the proportion of colonies surviving on plates containing (A) EMB or (B) ciprofloxacin (CIP) compared to plates without antibiotics. *▴*, the Mc^2^155 NJS20 control; ▪, the NJS20.1 *lsr2* transposon mutant; *⋄*, the NJS20.1c complemented strain. The mean and standard deviations of at least triplicate cultures of each strain are shown.

It has been shown previously that M. smegmatis strains overexpressing *iniA* are somewhat more resistant to EMB than controls [3]. We postulated that the NJS20.1 *lsr2* mutant would also be more resistant to EMB due to de-repression of *iniA*. NJS20.1 was indeed more resistant to EMB than either NJS20.w or NJS20.1c using the proportions method of susceptibility testing [10] (Figure 3A). Complementation restored EMB susceptibility to the level of the control strain. We tested all strains with ciprofloxacin in the same manner as the EMB assays to address the possibility that the *lsr2* activity is specific to cell wall antibiotics (Figure 3B). All strains were equally susceptible to ciprofloxacin at all concentrations, suggesting that the role of *lsr2* is limited to antibiotics that target the cell wall.

### Permeability Studies

Changes in colony morphology and antibiotic susceptibility can be associated with changes in cell wall permeability. Therefore, it was possible that the observed differences in P*_iniBAC_* induction could be due to increased entry of EMB into the cell. We measured the cell wall permeability of NJS20.1 to both hydrophilic and hydrophobic compounds by examining permeability to glycerol [carbonyl-C^14^] and chenodeoxycholic acid [carbonyl-C^14^], respectively. No change in the intracellular levels of either compound was noted in NJS20.1 compared to control NJS20.w (unpublished data), indicating that disruption of *lsr2* does not lead to substantial permeability changes.

### Microarray Analysis

**Figure 4:**
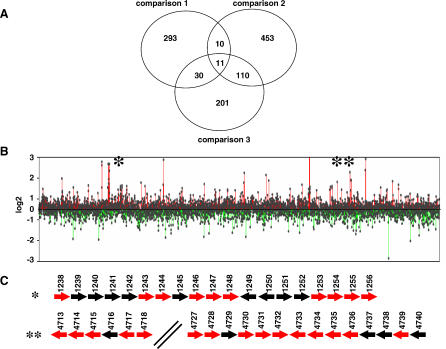
Whole M. smegmatis Genome Microarray Analysis (A) Venn diagram showing statistically significantly upregulated genes in each of the three experimental comparisons. Comparison 1, expression of NJS20 compared to that of the Δ*lsr2* strain NJS22; comparison 2, expression of NJS20 compared to that of NJS20 cultured in EMB; comparison 3, expression of NJS20 compared to that of NJS22 (both cultured in EMB). (B) Expression levels of all genes in the M. smegmatis chromosome in comparison 1. Each dot corresponds to a single open reading frame. (C) Magnification of two sections within the M. smegmatis genome (*, region 1; **, region 2 in [B]) that contain clusters of upregulated genes. All genes are represented by arrows; red arrows correspond to genes that are significantly upregulated in condition 1, black arrows correspond to genes that are not significantly upregulated in condition 1.

Microarray studies were performed to further investigate the role of *lsr2* in transcriptional regulation under baseline and antibiotic-inducing conditions. A new M. smegmatis Δ*lsr2* strain that contained a complete unmarked deletion of *lsr2* (strain NJS22) was created to perform these studies. Three different comparisons were made: Expression of NJS20 was compared to NJS22 expression to identify genes that were up- or downregulated by deletion of *lsr2* (comparison 1) (Table S1). NJS20 grown in 7H9 media was compared to NJS20 cultured with EMB to identify genes that were induced by EMB in wild-type M. smegmatis (comparison 2) (Table S2). NJS20 treated with EMB was compared to NJS22 treated with EMB to identify genes that were induced by EMB in a Δ*lsr2* background and to identify the EMB-induced genes that were further upregulated (“hyperinduced”) by *lsr2* deletion (comparison 3) (Table S3). All genes with statistically significant changes (*p* < 0.05) in gene expression were included in each analysis (Figure 4A). Comparison 1 revealed that 344 genes were upregulated and 286 genes were downregulated by *lsr2* deletion. The increased ratio of up- versus downregulated genes was even more remarkable when the analysis was restricted to genes with statistically significant expression changes of 1.5-fold (146 up- versus 103 downregulated) or 2-fold (41 up- versus 19 downregulated) and is consistent with our hypothesis that *lsr2* has broad and principally repressive effects on transcription. As predicted by the M. tuberculosis P*_iniBAC_* reporter assay, *M. smegmatis iniA* expression was significantly increased in NJS22 compared to NJS20, although absolute upregulation was only 1.3. Other broad categories of genes that were upregulated in condition 1 included genes involved in cell wall processes, metabolism, and transport (Table S1). Interestingly, stress response genes were not strongly represented among the genes upregulated in this comparison.

The microarray studies allowed us to search for DNA sequences that might represent binding sites for Lsr2 or an associated protein. However, no consensus sequences were identified in alignments of up to 400 bp upstream of the 15 most strongly induced genes. In contrast, these regions were found to be unusually adenine-thymine (AT)–rich (43.2% AT compared to a mean of 32.6% AT in the M. smegmatis genome). Mapping the induced and repressed genes over the entire chromosome revealed a number of chromosomal regions with large clusters of highly upregulated genes (Figure 4B). The upregulated genes within each cluster did not appear to comprise single operons (Figure 4C). As with the 20 most highly induced genes, the regions 400 bp upstream of the upregulated genes shown in Figure 4C were unusually AT-rich (41.4% AT for region 1 and 40.5% AT for region 2). These results are consistent with the hypothesis that *lsr2* encodes a protein with relatively non-specific rather than sequence-specific DNA-binding properties that preferentially binds to AT-rich sequences in a manner similar to that of some other histone-like proteins [11].

Our reporter studies had shown that the M. tuberculosis P*_iniBAC_* was upregulated by *lsr2* disruption in NJS20.1, induced by EMB in NJS20, and hyperinduced by EMB in NJS20.1. We examined conditions 1–3 to identify the complete complement of M. smegmatis genes that exhibited this expression pattern. As predicted, we found that *iniA* was significantly upregulated/induced in all three conditions. Interestingly, only ten other genes had similar expression patterns (Figure 4A; Table 2) (*p* = 0.0001 that this number of genes were not present in all three conditions by chance). This group was overrepresented by genes involved in cell wall biosynthesis, transport, or other cell wall functions, providing a link to *iniA,* which appears to encode for a pump-associated protein in M. tuberculosis [3]. One hundred and twenty-one genes were induced in both condition 2 and 3 (*p* = 0.0001), indicating that many of the genes that are induced by EMB in wild-type M. smegmatis are hyperinduced in a Δ*lsr2* background. These results support the hypothesis that *lsr2* is involved in controlling the level of expression of a subset of cell wall–active antibiotic-induced genes. Interestingly, only 21 genes were upregulated by condition 1 and induced in condition 2 (*p* = 0.81), while 41 were upregulated by condition 1 and induced in condition 3 (*p* = 0.0001). These results indicate that many of the genes controlled by *lsr2* are not related to EMB treatment, suggesting that *lsr2* is involved in the control of a broad range of cellular processes.

**Table 1:**
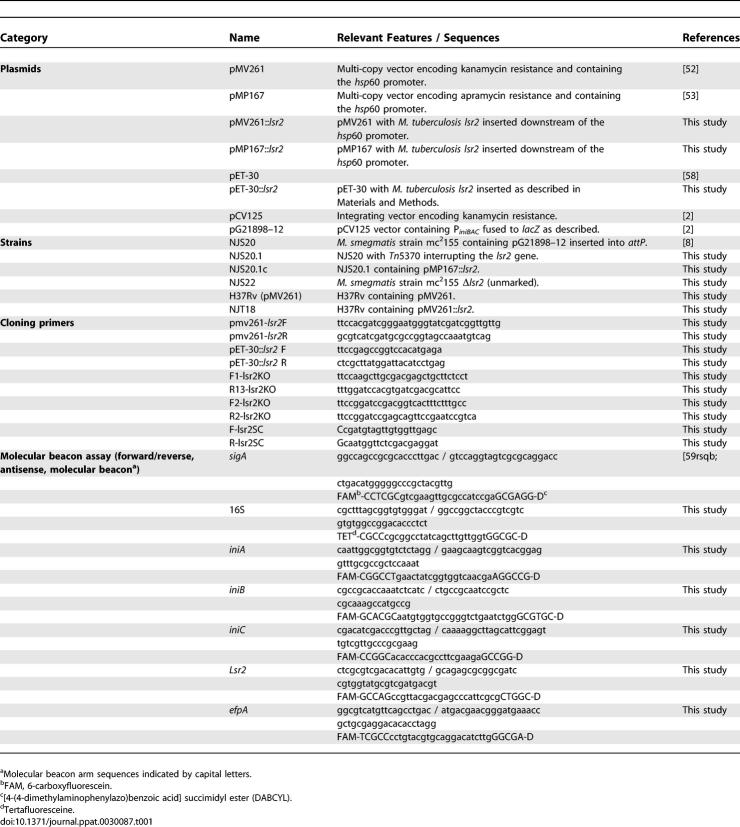
Plasmids, Strains, Primers and Molecular Beacons Used in This Study

**Table 2:**
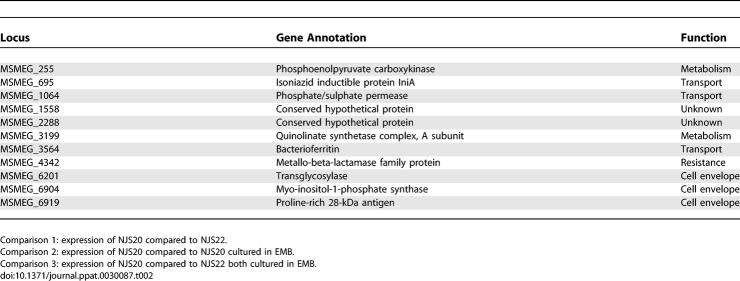
Genes Upregulated in All Three Comparisons

### Overexpression of *lsr2* Downregulates the *iniBAC* Operon and the *efpA* Gene in *M. tuberculosis*

**Figure 5:**
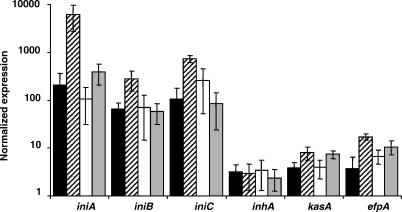
Effect of *lsr2* Overexpression on Expression of the *iniBAC* Operon, *inhA, kasA,* and *efpA* in *M. tuberculosis* The mRNA levels for wild-type *M. tuberculosis* H37Rv or of the *M. tuberculosis lsr2* overexpression strain NJT18 are shown with and without 24 h incubation in INH at a final concentration of 1.0 ug/ml. The mRNA levels are expressed as values that have been normalized to 16S mRNA levels in the same sample. Solid columns, H37Rv incubated in media without INH; dashed columns, H37Rv incubated with INH; open columns, NJT18 incubated in media without INH; gray columns, NJT18 incubated with INH. The mean and standard deviations of at least triplicate experiments and triplicate cultures of each strain and condition are shown.

The observation that *lsr2* participates in repression of the M. tuberculosis P*_iniBAC_* in M. smegmatis suggested that *lsr2* might perform a similar function in M. tuberculosis. We were unable to generate an M. tuberculosis Δ*lsr2* strain using allelic exchange methods to study this question directly [12]. Although this result cannot be taken as proof for gene essentiality, it is consistent with Himar1-based transposon mutagenesis studies, which indicate that *lsr2* is essential in M. tuberculosis H37Rv [13], and with the results of another transposon mutant screen in M. tuberculosis [14] in which Lsr2 insertions were only detected at the extreme 3-prime end of the gene (R. McAdam, personal communication). We then decided to study the effect of *lsr2* overexpression in M. tuberculosis in strain H327Rv by overexpressing *M. tuberculosis lsr2* using the multi-copy plasmid pMV261::*lsr2* (creating strain NJT18). NJT18 overexpressed *lsr2* approximately 70-fold, as confirmed by quantitative PCR (unpublished data). We cultured NJT18 and a wild-type H37Rv control strain containing the pMV261 vector (H37Rv (pMV261)) to mid log phase, incubated the cultures with INH at a final concentration of 1.0 ug/ml (or no antibiotic control) for 24 h, and then measured expression of *iniB, iniA,* and *iniC* by quantitative PCR. We found that overexpression of *lsr2* downregulated INH-mediated induction of the *iniBAC* genes in NJT18 compared to the H37Rv (pMV261) control (Figure 5). These results are consistent with our discovery that overexpression of *lsr2* in M. smegmatis repressed EMB-mediated induction of P*_iniBAC_,* and they confirm the role of *lsr2* in repressing gene expression in M. tuberculosis.

We examined the effect of *lsr2* overexpression on *kasA, efpA,* and *inhA* expression in order to determine whether *lsr2* acted specifically on the *iniBAC* operon or whether it had a more global effect (Figure 5). The *kasA* and *efpA* genes were examined because both genes are induced by INH. Furthermore, *efpA* has been annotated as a efflux pump [15], suggesting that it might have functions analogous to the *iniA*-associated pump. Expression of *inhA* was studied as a control because INH does not induce this gene. We found that *inhA* expression was not affected by *lsr2* overexpression. This indicates that *lsr2* overexpression does not cause generalized repression of all gene expression in M. tuberculosis. INH-mediated induction of *efpA* and *kasA* were modestly downregulated in the *lsr2* overexpression strain, although the downregulation of *kasA* induction did not appear to be statistically significant.

### Inactivation of *lsr2* Affects Lipid Composition

**Figure 6:**
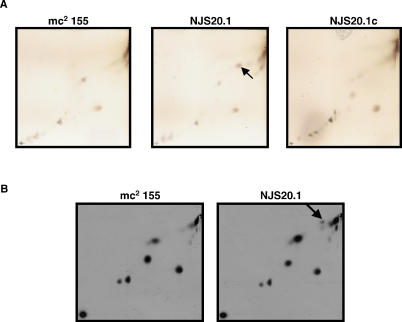
Two-Dimensional TLC Analysis of the Polar Lipids Extracted from M. smegmatis Strains (A) Two-dimensional TLC of polar lipid fractions from wild-type M. smegmatis Mc^2^155, *lsr2* transposon mutant NJS20.1, and complemented strain NJS20.1c. The solvent system is as follows: first dimension, chloroform/methanol/water (60/30/6); second dimension, chloroform/acetic acid/methanol/water (40/25/3/6). The spots were visualized with orcinol. The black arrow marks the spot present in the NJS20.1 mutant and not in either the control or the complemented strain. (B) The cells were grown to log phase and labeled with [1-^14^C]-acetate. The black arrow indicates the new lipid.

Inactivation of *lsr2* resulted in a remarkable change in colony morphology in M. smegmatis (Figure 1). A similar observation has been made previously by Chen et al. [9]. Colony morphology is often associated with a change in the cell wall structure [16–18]. Chen et al. noted that disruption of *lsr2* in M. smegmatis was associated with the disappearance of two apolar lipids. We analyzed the lipid composition of the NJS20.1 *lsr2* mutant in this study compared to that of the wild-type M. smegmatis Mc^2^155 strain by thin layer chromatography (TLC). The apolar and polar lipids from wild-type M. smegmatis and NJS20.1 were extracted and analyzed by one-dimensional and two-dimensional TLC. In contrast to the previous observations in [9], no difference was observed in the apolar fractions of the two strains (unpublished data). However, a new spot was observed in the polar fractions of NJT20.1 (Figure 6). Similar results were noted after [1-^14^C]-acetate labeling of actively dividing cells. These experiments suggest that this new compound is a glycolipid because it was visualized with orcinol, a reagent for detecting glycolipids, and the compound migrated like a glycolipid [19]. Furthermore, the compound integrated [1-^14^C]-acetate, indicating that it contains fatty acids. Transposon mutants may be complicated by polar effects, although this phenomenon can usually be controlled for by complementation experiments. We considered the possibility that the discrepancy between our results and those of Chen et al. could have been due to differences in the location of the transposon insertion. However, we obtained identical results when we repeated the lipid analysis with the Δ*lsr2* strain NJS22 (unpublished data).

### Lsr2 Is a Small Basic Protein with Histone-Like DNA-Binding Properties

**Figure 7:**
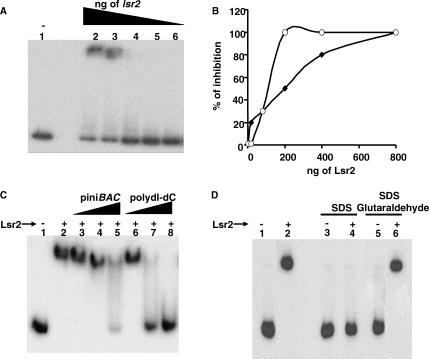
DNA Binding Properties of Lsr2 (A) EMSA assay of radiolabeled P*_iniBAC_* in the presence of Lsr2. Five fmole (0.7 ng) of P*_iniBAC_* were incubated with the following amounts of Lsr2: lane 1, 0 ng; lane 2, 400 ng; lane 3, 200 ng; lane 4, 100 ng; lane 5, 50 ng; lane 6, 25 ng. (B) Determination of the K_d_ of Lsr2-binding activity to P*_iniBAC_*. Five fmole (♦) and 50 fmole (○) of radiolabeled P*_iniBAC_* were incubated with the following amounts of Lsr2: 0 ng, 50 ng, 100 ng, 200 ng, 400 ng, and 800 ng; and then analyzed in EMSA assays. (C) Competition analysis. Fifty fmole of radiolabeled P*_iniBAC_* were incubated with 200 ng of Lsr2 where indicated. Different amounts of either cold P*_iniBAC_* or poly dI-dC were then added as competitors. Lane 1, no Lsr2 (all other lanes contain Lsr2); lane 2, no competitor; lane 3, 7 ng of P*_iniBAC_;* lane 4, 37 ng of P*_iniBAC_;* lane 5, 72 ng of P*_iniBAC_;* lane 6, 7 ng of poly dI-dC; lane 7, 37 ng of poly dI-dC; lane 8, 72 ng of poly dI-dC. (D) Specificity of DNA binding. Five fmole of radiolabeled P*_iniBAC_* were incubated with 200 ng of Lsr2 where indicated by a “+” followed by treatment with 0.5% SDS or SDS plus 0.1% glutaraldehyde where indicated. Lane 1, radiolabeled P*_iniBAC_* only; lane 2, Lsr2 added; lane 3, SDS only; lane 4, Lsr2 plus SDS; lane 5, SDS plus glutaraldehyde only; lane, 6 Lsr2 plus SDS and glutaraldehyde.

We prepared recombinant Lsr2 and then performed electrophoretic mobility shift assays (EMSAs) to characterize the ability of Lsr2 to specifically bind P*_iniBAC_*. A large mobility shift was observed when Lsr2 was incubated with a 227-bp PCR amplicon of the P*_iniBAC_* (Figure 7A). Titration of Lsr2 against two concentrations of P*_iniBAC_* (5 and 50 fmole) showed a dissociation constant (K_d_) of approximately 1 μM (Figure 7B). However, Lsr2 appeared to bind to DNA non-specifically, because a 200-bp PCR amplicon of the M. tuberculosis 16S rRNA gene produced similar mobility shifts and exhibited a similar K_d_ (unpublished data). Furthermore, competition analysis with unlabeled P*_iniBAC_* and poly dI-dC DNA (Figure 7C) (and 1 kb ladder; unpublished data) demonstrated an equal or better ability to compete for Lsr2 binding.

**Figure 8:**
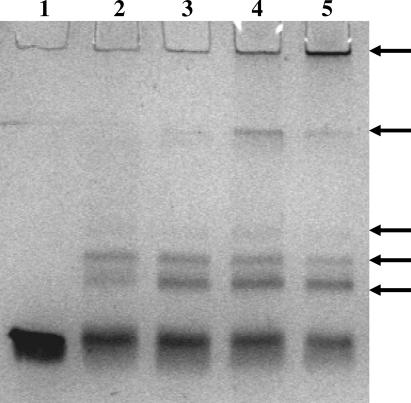
Progressive Lsr2 Oligomerization in the Presence of DNA Lsr2 (1 μg) was incubated with 0.5 μg of P*_iniBAC_* DNA in the presence of 0.1% of glutaraldehyde. Aliquots were analyzed at different time points on a Coomassie blue–stained SDS polyacrylamide gel. Lane 1, Lsr2 alone (control); lane 2, 1 min; lane 3, 2 min; lane 4, 5 min; lane 5, 10 min. Arrows indicate the location of the principal oligomer bands.

M. tuberculosis Lsr2 has a predicted mass of approximately 12 kDa and a pI of 10.69 These properties suggested that Lsr2 might have features in common with bacterial histone-like proteins [20] even though BLAST and iterative PSI-BLAST searches did not reveal any significant similarities. The mobility shifts observed in the EMSA assays indicated a complex that was much larger than would be expected by the association of a single 12-kDa Lsr2 molecule with its DNA target (Figure 7A). The formation of large protein–DNA complexes has also been reported with histone-like proteins [21–23]. We performed cross-linking studies between Lsr2 and P*_iniBAC_* DNA to test the specificity of the interaction between Lsr2 and DNA and to rule out the possibility that these complexes were caused by electrostatic binding artifacts. Treatment with 0.1% sodium dodecyl sulfate (SDS) caused the Lsr2–DNA complexes to dissociate, resulting in a loss of the original gel shift (Figure 7D). However, the shift was recovered by the addition of 0.1% of glutaraldehyde to the samples prior to SDS treatment. Lsr2 was then incubated with P*_iniBAC_* in the presence of glutaraldehyde for various times (Figure 8). Lsr2 multimers were detectable (in the form of multiple bands) as early as 1 min; longer incubation times produced very large complexes. Similar results were obtained after incubating Lsr2 with 1 kb ladder molecular weight marker DNA under similar conditions (unpublished data). These results demonstrate that Lsr2 directly interacts with a broad range of DNA sequences, resulting in the formation of large oligomeric complexes.

**Figure 9:**
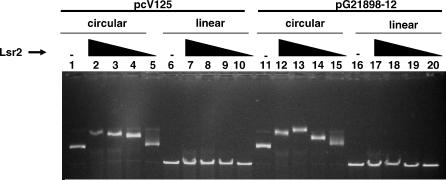
Preferential Binding of Lsr2 to Circular DNA: Agarose Gel Shift of Linearized versus Circular DNA Four hundred nanograms of either linear or circular pCV125 or pG21898–12 (pCV125 containing the P*_iniBAC_* sequence) were analyzed in the presence of different amounts of Lsr2 (600 ng of Lsr2 in lanes 2, 7, 12, and 17; 400 ng in lanes 3, 8, 13, and 18; 200 ng in lanes 4, 9, 14, and 19; and 100 ng in lanes 5, 10, 15, and 20). No Lsr2 was added to the control lanes 1, 6, 11, and 16. After 120 min of incubation at room temperature, the samples were analyzed on a 1% agarose gel.

Histone-like proteins have been reported to preferentially bind supercoiled DNA compared to linear DNA [20,22,24]. We incubated various amounts of Lsr2 with supercoiled and linear pCV125 vector to examine binding preference in an agarose-based gel shift assay. Identical experiments were also performed with a pCV125 vector containing the P*_iniBAC_* sequence (pG21898–12) to determine whether Lsr2 preferentially bound to P*_iniBAC_* under either of these conditions. A gel shift was only observed in the presence of the supercoiled plasmid. The results were similar whether or not the pCV125 plasmid contained P*_iniBAC_* sequences (Figure 9).

### Lsr2-Dependent DNA Protection and Inhibition of In Vitro Transcription

**Figure 10:**
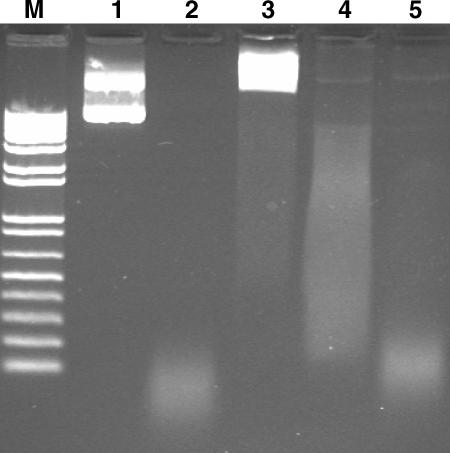
DNase I Protection Studies The ΦhX174 plasmid was incubated with Lsr2 where indicated and then treated with different concentrations of DNase I. Samples (except for lane 3) were treated with 6% SDS and 4 mg/ml protease K for 30 min at 37 °C before analysis on a 1% agarose gel. Lane 1, ΦhX174 alone; lane 2, ΦhX174 treated with 0.02 unit of DNase I; lane 3, ΦhX174 incubated with Lsr2 without DNase I treatment; lane 4, ΦhX174 incubated with Lsr2 followed by treatment with 0.02 unit of DNase I; lane 5, ΦhX174 incubated with Lsr2 followed by treatment with 1 unit of DNase I. M, DNA molecular marker.

Histones and histone-like proteins are typically able to protect DNA from degradation by DNase [20,23]. This property was tested in Lsr2 by first incubating ΦX174 DNA with Lsr2 and then treating the complex with 0.02 or 1.0 unit of DNase I for 1 min. Lsr2 was inactivated in each sample by treatment with protease K followed by boiling in SDS prior to analysis by gel electrophoresis to prevent a confounding gel shift of the Lsr2-treated DNA. We found that DNase I digested ΦX174 DNA into small fragments averaging less than 100 bp in size in the absence of Lsr2 pretreatment (longer periods of DNase I digestion completely digested the DNA). In contrast, DNase I activity was substantially inhibited by pretreatment with Lsr2 (Figure 10). Lsr2 pretreatment followed by digestion with 0.02 unit of DNase I produced a wide range of DNA fragments ranging from approximately 150 bp to the size of the supercoiled vector. Lsr2 pretreatment also conferred some protection against the activity of the higher concentration of DNase I, resulting in DNA fragments with an average size of approximately 150 bp (Figure 10), while this same concentration of DNase I completely digested the DNA sample in the absence of Lsr2 pretreatment (unpublished data). Heat-treated Lsr2 retained the ability to inhibit DNase, which is consistent with the known heat stability of histone-like proteins [25,26]. This protective effect could be the result of a histone-like interaction between Lsr2 and the DNA target of DNase. Alternately, Lsr2 could be inhibiting DNase due to a direct interaction between the Lsr2 and DNase proteins. DNase could not be co-eluted with Lsr2 bound to a nickel matrix when this experiment was performed to test for protein–protein interactions. These results suggest that Lsr2 does not interact directly with DNase.

**Figure 11:**
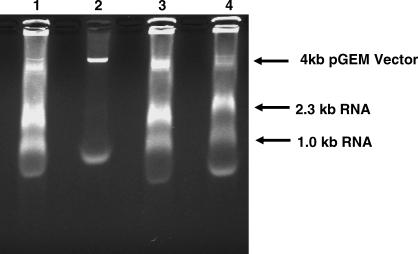
Effect of Lsr2 on In Vitro Transcription The pGEM plasmid was pre-incubated with different concentrations of Lsr2 where indicated. In vitro transcription was then performed either in transcription buffer or in buffer plus added imidazole (the Lsr2 elution) at the appropriate control concentration. Lane 1, pGEM with 200 ng of Lsr2; lane 2, pGEM with 600 ng of Lsr2; lane 3, pGEM with 40 nM imidazole (control for lane 1); lane 4, pGEM with 120 nM imidazole (control for lane 2).

We also tested the ability of Lsr2 to inhibit transcription in vitro as has been reported for other histones and histone-like proteins [27,28]. We used a standard in vitro T7 promoter–based transcription assay of a pGEM vector for these experiments because Lsr2 did not appear to bind specifically to M. tuberculosis DNA sequences. In the absence of Lsr2 pretreatment, transcription of the pGEM vector produced the expected 1.0- and 2.3-kb mRNA transcripts (imidazole was added to these reactions to control for the presence of imidazole in the buffer containing Lsr2). The expected mRNA transcripts were also present in transcription reactions containing 200 ng of Lsr2; however, 600 ng of Lsr2 completely inhibited transcription (Figure 11). In order to confirm that transcription was not being inhibited by non-specific effects of an added protein, we repeated these experiments after identical amounts of M. tuberculosis ESAT 6 protein were added to the transcription reaction. In contrast to Lsr2, ESAT 6 did not inhibit transcription (unpublished data).

### Effect of Lsr2 on Topoisomerase I–Dependent Supercoil Relexation

**Figure 12:**
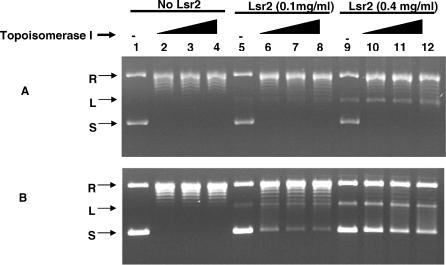
Effect of Lsr2 on Topoisomerase I Activity Different amounts of topoisomerase I were added to 200 ng of supercoiled ΦX174 RT DNA. Lanes 1, 5, and 9, no topoisomerase; lanes 2, 6, and 10, 2.4 units of topoisomerase; lanes 3, 7, and 11, 6 units of topoisomerase; lanes 4, 8, and 12, 12 units of topoisomerase. Lsr2 at 0.1 mg/ml or 0.4 mg/ml was added to the reaction either simultaneously with the topoisomerase or after 30 min incubation with topoisomerase alone. The reaction was then treated with 6% SDS and 4 mg/ml proteinase K analyzed on a 0.7% agarose gel and then stained with ethidium bromide. (A) Topoisomerase I and Lsr2 were incubated simultaneously with ΦX174. (B) Lsr2 was added 30 min after the topoisomerase incubation. L, linearized plasmid; R, relaxed plasmid; S, supercoiled plasmid.

DNA relaxation assays are often used to characterize the ability of histones and histone-like proteins to introduce supercoils into relaxed DNA in the presence of topoisomerase I [7,22]. We relaxed supercoiled ΦX174 DNA with topoisomerase I, then added Lsr2 and measured its ability to re-introduce supercoils. Lsr2 produced a small degree of additional supercoils to the relaxed DNA in this assay, consistent with histone-like activity. A small amount of linear DNA was also produced, suggesting that Lsr2 has nuclease properties (Figure 12A). These results could indicate that Lsr2 has only a modest histone-like ability to introduce supercoils into DNA. However, it was possible that Lsr2 also inhibited the interaction between topoisomerase I and the DNA target, which is also necessary for the introduction of supercoils in this assay. To differentiate between these two possibilities, we repeated the DNA relaxation assay, this time simultaneously adding Lsr2 and topoisomerase I to the supercoiled ΦX174 DNA. We found that topoisomerase I produced substantially less relaxed DNA when it was co-incubated with Lsr2, especially when higher amounts of Lsr2 were used (Figure 12B). Inhibition of topoisomerase I appears to be a novel activity that has not been reported for other bacterial histone-like proteins.

## Discussion

We have shown that Lsr2 is a histone-like protein with broad downregulatory and (to a lesser extent) upregulatory activity in M. smegmatis. The *lsr2* gene also appears to regulate the degree of EMB-mediated induction in a large set of genes that are induced in wild-type M. smegmatis by EMB. In *M. tuberculosis, lsr2* appears to downregulate antibiotic-mediated induction of the *M. tuberculosis iniBAC* and *efpA* genes. Our results suggest that *lsr2* has a role in regulating the drug tolerance phenotype that is associated with *iniA* overexpression in M. smegmatis and BCG. Lsr2 is also likely to have other regulatory functions, including those associated with cell wall biosynthesis, transport, and responses to antibiotic treatment.

The *lsr2* genes of *M. leprae, M. tuberculosis,* and *M. smegmatis* share an unusually high degree of homology (87% identity and 91% similarity for M. leprae compared to *M. tuberculosis;* 87% identity and 90% similarity for M. smegmatis compared to M. tuberculosis), suggesting an important biological role in these species. Lsr2 was first reported to be one of the major seroreactive proteins in M. leprae patients, and was further characterized as a seroreactive protein in both leprosy and tuberculosis [29,30–32]. Our discovery that Lsr2 binds DNA may explain the high immunoreactivity observed in these prior studies. We postulate that Lsr2 exists as a complex with mycobacterial DNA in extracellular fluid, where it serves as a potent adjuvant by simulating TLR-9 in macrophages and dendritic cells [33]. However, the importance of this immune response in immunopathogenesis or host immunity to tuberculosis or leprosy remains unclear. The *lsr2* gene has been previously reported to be induced by a number of stress conditions, including starvation, heat shock, and INH treatment [34–36]. Lsr2 production was also found to be induced in cultures supplemented with iron [37]. The association between induction of *lsr2* expression and these stress responses may provide additional clues to its role in cellular regulation.

Histones have been shown to broadly regulate transcription in eukaryotic cells through their influence on chromosomal topology [20]. The histone-like proteins of bacteria represent a diverse group of molecules that share the common property of small size and strong positive charge. Bacterial histone-like proteins have been associated with regulation of various cell stresses or responses to environmental changes [7,38,39]. Some histone-like proteins in Escherichia coli have been shown to directly affect antibiotic resistance by controlling expression of efflux pumps [40]. Disruption of *hupA* (one of the two genes encoding the HU protein) in E. coli K-12 had recently been shown to cause morphological changes similar to those we observed in NJS20.1 and NJS22 [41]. Very little is known about histone-like proteins in mycobacteria. The *M. smegmatis hlp* gene encodes a histone-like protein that is induced by cold-shock [42] and anaerobic-induced dormancy [43]. The *hlp* gene was also found to be important for invasion of M. leprae into peripheral nerves, and it has been hypothesized that it acts as an adhein during mycobacterial infections [44]. MDP1, the M. tuberculosis and BCG homolog of *hlp,* has recently been shown to bind DNA and inhibit transcription in vitro in a manner similar to that of Lsr2. MDP1 is also induced in stationary phase cultures, and appears to participate in the binding of M. tuberculosis to alveolar epithelial cells [45–47]. However, unlike *lsr2,* MDP1 has homologies to other histone-like proteins, such as *hlp* and the E. coli HU protein; furthermore, the regulatory roles (if any) of MDP1, and other histone-like proteins in *M. tuberculosis,* are not known.

In addition to its size and pI, Lsr2 shares many properties with other bacterial histone-like proteins. Our microarray studies identified clusters of genes with AT-rich 5-prime untranslated sequences that were induced in the Δ*lsr2* strain. This finding closely parallels the activity of the histone-like protein H-NS, which transcriptionally silences clusters of laterally acquired genes in *Salmonella* by binding to AT-rich sequences [11]. We showed that Lsr2 forms large multimeric complexes with DNA (with a preference to supercoiled forms), protects against DNase I treatment, and introduces a modest degree of supercoiling into relaxed plasmids, properties consistent with histone-like proteins. Lsr2 also appears to inhibit in vitro transcription and topoisomerase I. Co-elution studies did not detect any interactions between Lsr2 and DNase, suggesting that Lsr2 exerts its suppressive effect by interacting with DNA rather than by directly inhibiting proteins. Given the other similarities of Lsr2 to histone-like proteins, it is likely that Lsr2 inhibits RNA polymerase and topoisomerase activity by causing topological changes to the DNA targeted by these enzymes. This inhibition may be related to the ability of Lsr2 to form large oligomeric complexes with DNA. It is possible that the activity of Lsr2 is modulated in vivo by other cellular proteins and by local variations in chromosomal sequences; however, this remains to be determined. A PSI-BLAST analysis of the Lsr2 sequence reveals a nuclease motif, which is consistent with the weak nuclease activity that was noted in some of our experiments and may be related to its function. The Lsr2 sequence is unique, exhibiting no significant similarities to any histone-like protein. Thus, Lsr2 represents a novel class of histone-like proteins.

Our discovery that Lsr2 is involved in regulating a subset of INH- and EMB-inducible genes suggests at least one significant function. Investigations of the cellular responses to antibiotic treatments (as distinct from investigations of antibiotic resistance mechanisms) are in their infancy. Although deletion of the histone-like protein gene *hns* in E. coli was found to de-repress expression of multi-drug transporters related to TolC and confer multi-drug resistance [40], this work represents the first investigation to our knowledge of the regulation of antibiotic-induced genes in M. tuberculosis. Studies of cellular responses to antibiotics may be crucial to understanding the mechanisms by which bacteria survive or die in the presence of antibiotics. For example, we previously demonstrated that the *iniBAC* genes are induced by INH, EMB, and a number of other antibiotics that act by inhibiting cell wall biosynthesis in M. tuberculosis [8]. Induction of *iniA* was shown to confer multi-drug tolerance through the action of a multi-drug resistance–like pump [3]. Antibiotic tolerance can occur through other mechanisms such as overproduction of various inhibitors, enzymes, and regulatory proteins in non-mycobacterial bacteria [4,5,48–50]. It is possible that a multi-functional *lsr2* also regulates these and other pathways in mycobacteria.

Deletion analysis of *lsr2* in M. tuberculosis would be particularly useful for studies of its function. Unfortunately, we have been unable to delete *lsr2* from this species, although deletion was easily accomplished in M. smegmatis. The *lsr2* gene may be essential in M. tuberculosis. *lsr2* was suggested to be essential by Himar1-based transposon mutagenesis [13], and all of the *lsr2* transposon mutants characterized by McAdam et al. [14] contained insertions at the extreme 3-prime end of the gene where the transposon would be unlikely to affect functional capacity. If confirmed by future studies, the finding that this gene is essential is consistent with our hypothesis that *lsr2* regulates important cellular pathways in M. tuberculosis.

The *lsr2* gene has been found to be essential for biofilm formation in M. smegmatis. This study also showed that an *M. smegmatis lsr2* transposon mutant had altered colony morphology and contained two previously unidentified apolar lipids that were novel mycolate-containing compounds [9]. We found the same altered colony morphology but did not detect any novel apolar lipids in our analysis of the *M. smegmatis (lsr2::Tn5370)* strain NJS20.1 or in the Δ *lsr2* strain NJS22. However, we did detect a new compound, possibly a glycolipid, in the polar fraction of both NJS20.1 and NJS22. The dissimilarity between these two lipid analyses could be due to a difference in the way the strains were grown or harvested for the TLC analysis. Despite the apparent contradiction between the two studies, both investigations suggest that *lsr2* is involved in regulating a wide range of cellular processes.

In summary, *lsr2* encodes a histone-like DNA-binding protein that appears to be essential for controlling responses to certain types of antibiotic stress. Lsr2 has also been linked to other types of stress responses and other cellular functions in mycobacteria. An improved understanding of the role of *lsr2* and of the stress responses associated with this gene may provide important insights into the mechanisms of action of antibiotics and the way that mycobacteria adapt to certain types of stresses such as antibiotic treatment. This knowledge could in turn be used to design more effective antibiotic treatments for both drug-susceptible and drug-resistant M. tuberculosis.

## Materials and Methods

### Bacterial strains and culture conditions.

E. coli DH5α was the host for all plasmid constructions. Experiments with M. smegmatis either used Mc^2^155 or, in the case of the transposon mutagenesis experiments, NJS20, a Mc^2^155 strain containing the pG21898–12 plasmid integrated into Mc^2^155 chromosome at *attP* (Table 1) [8]. The pG21898–12 plasmid is a reporter construct that contains the M. tuberculosis P*_iniBAC_* fused to *lacZ* [8]. Experiments with M. tuberculosis used strain H37Rv. E. coli was cultured at 37 °C in Luria-Bertani medium with the addition of hygromycin B (200 μg/ml; Sigma, http://www.sigmaaldrich.com) or kanamycin (40 μg/ml; Sigma) where appropriate. M. smegmatis strains were grown at 37 °C on a rotary shaker in Middlebrook 7H9 medium (Difco, http://www.vgdusa.com/DIFCO.htm) containing 0.05% Tween 80, 0.02% glycerol, 10% ADC (Sigma) [51], and 25 ug/ml kanamycin or 40 μg/ml apramycin as appropriate. Transposon mutants of NJS20 were cultured in the presence of hygromycin B (50 μg/ml). M. tuberculosis strains were cultured at 37 °C on a rotary shaker in Middlebrook 7H9 medium (Difco) containing 0.05% Tween 80, 0.02% glycerol, and 10% ADC with 12.5 μg/ml kanamycin added as appropriate.

### Plasmid construction.

The complete *lsr2* gene (nucleotides 4040981–4041319) was amplified by PCR from H37Rv chromosomal DNA using primers pMV261-*lsr2*F and pMV261-*lsr2*R (Table 1), digested with PvuII and ClaI, and then cloned into pMV261 [52] (which encodes for kanamycin resistance) to create pMV261::*lsr2,* or into pMP167 [53] (which encodes for apramycin resistance) at the PstI/ClaI sites to create pMP167::*lsr2* (Table 1). *M. tuberculosis lsr2* ORF (nucleotides 4040981–4041319) was inserted into the NdeI/XhoI sites of pET-30, creating pET::*lsr2*. The pET::*lsr2* plasmid was transformed into BL-21 E. coli competent cells. Plasmids pCV125, pG21898–12, and pBluescript have been described elsewhere [8].

### Transposon mutagenesis.

Transposon mutagenesis was performed in the *M. smegmatis iniBAC* promoter reporter strain NJS20 as described [54] using the minitransposon vector pJSC84, which contains inverted repeats flanking a hygromycin cassette [54]. The construct was packaged in a TM4 temperature-sensitive phage, and transfected into NJS20. Cells were grown in 7H9 to mid log phase, prewarmed to the non-permissive temperature of 37 °C, and then mixed with 10^10^ pfu/ml (multiplicity of infection 10). The cell–phage mixture was incubated at the non-permissive temperature of 30 °C for 30 min, plated on 7H10 agar containing IPTG and β-galactosidase, and then incubated at 37 °C for 2–3 d.

### Creating an unmarked deletion of *lsr2* in M. smegmatis.

Strain NJS22, an M. smegmatis strain containing a complete unmarked deletion of *lsr2,* was created using the *sacB* counter selection method as described previously [55]. Briefly, DNA sequences from 6154142 to 6155817 and from 6156110 to 6157718 in M. smegmatis Mc^2^155 were PCR amplified using primer pairs F1-lsr2KO - R13-lsr2KO and F2-lsr2KO - R2 lsr2KO, respectively (Table 1), and cloned into the p2NIL vector [55], followed by insertion of a PacI cassette containing *sacB* and *lacZ.* Blue colonies containing single crossover events were identified on X-gal/kanamycin media. Double crossover events from these blue colonies were selected on 2% sucrose/X-gal media. Deletion mutants were confirmed by real-time PCR for the *lsr2* gene using primers Flsr2SC and Rlsr2SC (Table 1).

### β-galactosidase assays.

Assays were performed as described [8] using *o*-nitrophenyl-β-D-galactopyranoside (4 mg/ml; Sigma) to detect the presence of β-galactosidase activity. β-galactosidase units were calculated using the formula 1,000 × OD_420_/time (minutes) × 0.5 × OD_590_.

### Generation of probes for microarray experiments.

cDNA probes for microarray experiments were generated as previously described [56]. One microgram of mRNA in a mixture containing 6 μg of random hexamers (Invitrogen, http://www.invitrogen.com), 0.01 M dithiothreitol, an aminoallyl-deoxynucleoside triphosphate mixture containing 25 mM each dATP, dCTP, and dGTP, 15 mM dTTP, and 10 mM amino-allyl-dUTP (aa-dUTP) (Sigma), reaction buffer, and 400 units of Powerscript reverse transcriptase (Clontech, http://www.clontech.com) was incubated at 42 °C overnight. The RNA template then was hydrolyzed by adding NaOH and EDTA to a final concentration of 0.2 and 0.1 M, respectively, and incubating at 65 °C for 15 min. Unincorporated aa-dUTP was removed with a Minelute column (Qiagen, http://www.qiagen.com). The probe was eluted with a phosphate elution buffer (4 mM KPO_4_ [pH 8.5], in ultrapure water), dried, and resuspended in 0.1 M sodium carbonate buffer (pH 9.0). To couple the amino-allyl cDNA with fluorescent labels, nhs-Cy3 or nhs-Cy5 (Amersham, http://www.amersham.com) was added at room temperature for 2 h. Uncoupled label was removed using the Qiagen Minelute PCR purification.

### Microarray hybridization, scanning, image analysis, normalization, and analysis.

Microarray studies of each condition were performed with three separate RNA samples obtained from separate cultures using a dye-flip protocol (two microarrays for each RNA sample). Epoxy- or aminosiline-coated slides were prehybridized in 5x SSC (1x SSC is 0.15 M NaCl plus 0.015 M sodium citrate) (Invitrogen), 0.1% SDS, and 1% bovine serum albumin at 42 °C for 60 min. The slides then were washed at room temperature with distilled water, dipped in isopropanol, and spun dry. Equal volumes of the appropriate Cy3- and Cy5-labeled probes were combined, dried, and then resuspended in a solution of 40% formamide, 5x SSC, and 0.1% SDS. Resuspended probes were denatured to 95 °C prior to hybridization. The probe mixture then was added to the microarray slide and allowed to hybridize overnight at 42 °C. Hybridized slides were washed sequentially in solutions of 1x SSC and 0.2% SDS, 0.1x SSC and 0.2% SDS, and 0.1x SSC at room temperature, then dried, and scanned with an Axon GenePix 4000 scanner (http://www.moleculardevices.com). Individual TIFF images from each channel were analyzed with TIGR Spotfinder (http://www.tm4.org). Microarray data were normalized by Iterative Log Normalization using TIGR MIDAS software (http://www.tm4.org). Filtration of data was done in Microsoft Excel. Median spot intensity values were used to calculate the log_2_ ratio and fold change. Genes considered significantly differentially expressed by microarray analysis were identified by a one class *t*-test analysis comparing flip dye pairs of microarray experiments. The *p*-value was calculated based on permutation with random sampling using an overall alpha value ≤ 0.05. Unequal group variance was assumed by Welch assumption.

### Measurements of *lsr2* mutant cell wall permeability.

Mid log phase cultures of M. smegmatis strains were grown in 7H9 media to an OD_600_ of 0.8. Either glycerol [carbonyl-C^14^] (Amersham Phar-macia Bio, http://www.gelifesciences.com) or chenodeoxycholic acid [carbonyl-C^14^] (American Radiolabeled Chemical, http://www.arcincusa.com) were added at a concentration of 0.5 μCi/ml and 2.0 μCi/ml, respectively, to the cultures to test the permeability of glycerol and chenodeoxycolic acid. The cultures were again incubated at 37 °C with gentle shaking. At different time points, 200 ml of each culture were removed and applied to glass fiber filters (Schleicher & Schuell, http://www.whatman.com). A vacuum was applied using a vacuum filtration unit. Samples were washed twice with water and once with ethanol. Filters were dried at room temperature for 10 min and the amount of radioactivity was measured by scintillation counting.

### Expression and purification of recombinant Lsr2.

Purified recombinant Lsr2 protein was obtained by fusing the *M. tuberculosis lsr2* gene with the 6-histidine tag at the NH2 terminus using the pET-30 vector. The 12-kDa–tagged rLsr2 protein was purified to near-homogeneity by nickel affinity chromatography (Amersham Pharmacia Bio) under non-denaturing conditions using an imidazole gradient. Eluted fractions were assessed in a 12% polyacrylamide gel, revealing a single band of the expected molecular weight. Western blot analysis using anti–His tag antibody (Amersham Pharmacia Bio) confirmed the presence of a single band corresponding to Lsr2.

### Electrophoretic mobility shift assay.

The 227-bp promoter region of P*_iniBAC_* (nucleotides 409379–409560) was amplified by PCR reaction using α-dCTP in the reaction. Radiolabeled P*_iniBAC_,* poly dI-dC, or 1 kb ladder were incubated with different concentrations of Lsr2 protein for 20 min on ice in a 10-μl reaction cocktail containing 20 mM Tris-HCl (pH 7.0), 0.01% BSA, 2 mM DDT, and 10 mM NaCl. Protein–DNA complexes were resolved by electrophoresis through a 5% polyacrylamide gel for 1 h at 4 °C and then examined by autoradiography.

### Protein–protein cross-linking.

Different amounts of recombinant Lsr2 were incubated with P*_iniBAC_* DNA in a mixture containing 20 mM Tris-HCl (pH.7.0), 0.01% BSA, 2 mM DDT, and 10 mM NaCl. Cross-linking was performed by the addition of 0.1% of glutaraldehyde (Sigma) to the mix. Aliquots were taken and loaded onto a 10% polyacrylamide gel at different time points.

### DNase I protection.

ΦX174 (Promega, http://www.promega.com) plasmid DNA (0.5 μg) was incubated for 20 min on ice with or without Lsr2 (200 ng) in a final volume of 50 ul. Either 0.02 or 1 unit of DNase I (New England Biolabs, http://www.neb.com) was then added, and the mixture was incubated at 37 °C for 1 min. DNase was inactivated by incubating at 75 °C for 15 min. The samples were then treated with 6% SDS and 4 mg/ml of protease K for 30 min at 37 °C, and then analyzed on a 1% agarose gel.

### Co-elution studies.

Recombinant Lsr2 (1 ug) was incubated with a nickel Sepharose matrix (GE Healthcare, http://www.gehealthcare.com) for 1 h at room temperature. The unbound Lsr2 was removed by washing the matrix three times with 0.1 M PBS, 20 mM MgCl_2_, and 20 mM NaCl. DNase I (5 ug; New England Biolabs, http://www.neb.com) was added to the nickel matrix–Lsr2 and incubated overnight at 4 °C. After washing using 0.1 M PBS, 20 mM MgCl_2_, and 20 mM NaCl, Lsr2 was eluted from the nickel matrix using 0.1 M PBS, 20 mM MgCl_2_, 20 mM NaCl, and 20 mM imidazole. Eluted proteins were assessed in each fraction by Coomassie blue–stained SDS-PAGE.

### In vitro transcription.

Briefly, 0.5 μg of pGEM was incubated with either control imidazole buffer or either 200 or 600 ng of Lsr2 in a final volume of 20 ul, followed by in vitro transcription according to the manufacturer's recommendation using the Riboprobe in vitro Transcription Systems (Promega). Samples were treated with 6% SDS and 4 mg/ml protease K for 30 min at 37 °C and then analyzed on a 1% agarose gel.

### DNA relaxation assay.

The assay was carried out using a standard protocol [22]. Briefly, 200 ng of ΦX174 RT DNA (Promega) was incubated in the presence of different units (2–12 units) of topoisomerase I (Invitrogen), with and without Lsr2, and incubated at 37 °C for 30 min. After incubation, 6% SDS and proteinase K (4 mg/ml; Invitrogen) were added to the mixture, which was incubated for 15 min at 37 °C. Samples were run on a 0.7% agarose gel and then stained with ethidium bromide (Sigma) for analysis.

### Gene expression in the presence and absence of INH.

Total RNA was extracted from M. tuberculosis strains. cDNA synthesis and quantitative PCR with molecular beacons were performed as described previously [57]. The molecular beacons and primers used to study expression of *kasA* and *inhA* were described previously [57], and the molecular beacons and primers used to study expression of *lsr2, iniB, iniA, iniC,* and *efpA* are shown in Table 1.

### Lipid extraction.

M. smegmatis strains (50 ml) were grown in Mueller-Hinton broth (Difco) at 37 °C up to late log phase. The cultures were spun and the cell pellets were washed once with PBS buffer. After discarding the supernatants, the cell pellets were lyophilized. The dried pellets were treated as described previously [19]. Briefly, the cell pellet (0.2 g) was resuspended in methanol/0.3% aqueous NaCl solution (2 ml; 10/1, v/v) and the suspension was extracted twice with petroleum ether (1 ml) for 15 min at room temperature. The petroleum ether phases were combined and dried under nitrogen to yield the apolar lipid fraction, which was resuspended in dichloromethane. The methanol/saline fraction was heated at 100 °C for 5 min, cooled, and treated with chloroform/methanol/0.3% aqueous NaCl solution (2.5 ml; 9/10/3, v/v/v) at room temperature for 45 min. After centrifugation, the solvent fraction was removed and the residue was extracted with chloroform/methanol/0.3% aqueous NaCl solution (1 ml; 5/10/4, v/v/v) at room temperature for 15 min and spun (3000*g,* 2 min). The solvent fractions were combined, mixed with chloroform/0.3% aqueous NaCl solution (3 ml, 1/1, v/v), vortexed, and spun. The upper phase was discarded and the lower phase was dried under nitrogen to yield the polar lipids fraction, which was solubilized in chloroform/methanol (2/1, v/v).

The lipid fractions were analyzed by one-dimensional and two-dimensional TLC using silica gel 60F-254 TLC plates (Alltech, http://www.alltech.com). The following solvent systems were used to run one-dimensional TLC: hexane/ethyl acetate (9/1, v/v); hexane/ethyl acetate (1/1, v/v); ethyl acetate; chloroform/methanol (95/5, v/v); chloroform/methanol/water (90/10/1, v/v/v); and chloroform/methanol/water (60/30/6, v/v/v). For two-dimensional TLC, the solvent systems used were as follows: either first dimension, chloroform/methanol/water (100/14/0.8, v/v/v), second dimension, chloroform/acetone/methanol/water (50/60/2.5/3, v/v/v/v); or first dimension, chloroform/methanol/water (60/30/6, v/v/v), second dimension, chloroform/acetic acid/methanol/water (40/25/3/6, v/v/v/v). The lipids were visualized by spraying with a 10% sulfuric acid solution in ethanol or with an orcinol solution (Alltech).

### Radiolabeling of lipids with [1-^14^C]-acetate.

The M. smegmatis strains were grown in Middlebrook 7H9 supplemented with 0.2% glycerol, 10% ADS enrichment, and 0.2% Tween 80 to log phase (OD_600_ = 0.8), and labeled with [1-^14^C]-acetate (15 μCi) for 1 h. After centrifugation, the lipids were extracted as described above.

## Supporting Information

Table S1 Genes Upregulated (1.4 MB XLS)

Table S2 Genes Downregulated (161 KB XLS)

Table S3 Genes Downregulated (114 KB XLS)

## Abbreviations

AT: adenine-thymine
EMB: ethambutol
EMSA: electrophoretic mobility shift assay
INH: isoniazid
P_*iniBAC*_: *iniBAC* promoter
SDS: sodium dodecyl sulfate
TLC: thin layer chromatography
